# Impact of Myeloid p38α/MAPK on Orthodontic Tooth Movement

**DOI:** 10.3390/jcm11071796

**Published:** 2022-03-24

**Authors:** Christian Kirschneck, Hendrik Nusser, Jonathan Jantsch, Peter Proff, Agnes Schröder

**Affiliations:** 1 Department of Orthodontics, University Hospital Regensburg, 93053 Regensburg, Germany; christian.kirschneck@ukr.de (C.K.); hendrik.nusser@stud.uni-regensburg.de (H.N.); peter.proff@ukr.de (P.P.); 2 Institute of Clinical Microbiology and Hygiene, University Hospital Regensburg, 93053 Regensburg, Germany; jonathan.jantsch@ukr.de

**Keywords:** p38 mitogen-activated protein kinases, myeloid cells, orthodontic tooth movement

## Abstract

*Objectives*: Myeloid p38α/MAPK regulate and coordinate osteoclastogenesis. The present study was conducted to investigate the role of myeloid p38α/MAPK during orthodontic tooth movement. *Methods*: Orthodontic tooth movement was performed in wildtype and *p38α*^Δmyel^ mice lacking p38α/MAPK expression in myeloid cells. First, bone parameter as well as osteoblast and osteoclast number were determined in tibiae. RNA was isolated from the untreated and orthodontically treated maxillary jaw side and expression of genes involved in inflammation and bone remodelling were analysed. Finally, periodontal bone loss, alveolar bone density and extent of orthodontic tooth movement were assessed. *Results*: Bone density was increased in *p38α*^Δmyel^ mice compared to wildtype mice in tibiae (*p* = 0.043) and alveolar bone (*p* = 0.003). This was accompanied by a reduced osteoclast number in tibiae (*p* = 0.005) and TRAP5b in serum (*p* = 0.015). Accordingly, expression of osteoclast-specific genes was reduced in *p38α*^Δmyel^ mice. Extent of tooth movement was reduced in *p38α*^Δmyel^ mice (*p* = 0.024). This may be due to the higher bone density of the p38αΔmyel mice. *Conclusions*: Myeloid p38α/MAPK thus appears to play a regulatory role during orthodontic tooth movement by regulating osteoclastogenesis.

## 1. Introduction

Orthodontic tooth movement is critically dependent on remodelling processes in the alveolar bone. The application of an orthodontic force to a tooth creates pressure and tension zones in the periodontal ligament. Osteoclastic bone resorption processes predominantly take place at the pressure zones, while new bone is formed by osteoblasts at the tension side [1,2]. These bone remodelling processes are regulated by the cells in the periodontal ligament through the release of proinflammatory mediators such as cytokines and chemokines. Among them are mainly periodontal ligament fibroblasts [3,4], but also immune cells such as T lymphocytes [5] and macrophages [6,7]. Previous studies have already shown that macrophages react to mechanical forces with changed secretion of inflammatory mediators such as tumor necrosis factor (TNF), interleukin-6 (IL6) and prostaglandin E2 [6,8]. Cells of the immune system thus seem to play an important regulatory role for orthodontic tooth movement.

Numerous signal transduction pathways, such as the mitogen-activated kinase pathway (MAPK), also play a mediating role in orthodontic tooth movement [1,9]. The MAPK signalling pathway consists of a number of Ser/Thr kinases, such as extracellular signal-regulated kinase (ERK)1/2, c-Jun N-terminal kinase (JNK), ERK5 subfamilies, and p38 [10,11]. Playing a regulatory role in both osteoblast and osteoclast differentiation, the p38/MAPK pathway is closely associated with bone resorption and formation [12,13]. In addition, p38/MAPKs are activated by numerous growth factors and cytokines such as receptor activator of NFkB ligand (RANKL) and TNF [11,14], which are also increasingly released during orthodontic tooth movement [1,4]. Cell culture studies have also shown that mechanical stress activates ERK1/2 and p38/MAPK in periodontal ligament fibroblasts [15,16,17]. Additionally, animal studies showed enhanced phosphorylation of ERK1/2 and p38/MAPKs in periodontal tissues of orthodontically treated teeth [9]. 

The role of myeloid p38α/MAPK during orthodontic tooth movement has not been investigated yet. Since p38/MAPK play a crucial role in bone remodelling, the hypothesis of this work is that myeloid p38α/MAPK play a regulatory role during orthodontic tooth movement by affecting alveolar bone remodelling. Therefore, in this study, LysM^Cre^p38α^fl/fl^ mice were examined, in which p38α, an essential p38 isoform, was deleted in monocytes. The effects of the deletion on bone parameters in the tibia and alveolar bone, expression of genes associated with orthodontic tooth movement in the periodontal tissue, periodontal bone resorption, and the extent of orthodontic tooth movement were investigated.

## 2. Materials and Methods

### 2.1. Study Design and Experimental Animals

Animal experiments were conducted in accordance with the German Animal Protection Act and approval of the responsible authorities (ID: 55.2-2532-2-567). To avoid unnecessary suffering, mice welfare was checked on a daily basis (AS) and termination criteria were defined and approved by relevant authorities. Mice were housed in a conventional animal laboratory (21 °C; 12-h light–dark rhythm, free access to water and standard diet V1535, ssniff, Soest, Germany). Eight-week-old LysM^WT^p38α^fl/fl^ (WT; *n* = 21) and LysM^Cre^p38α^fl/fl^ (p38α^Δmyel^; *n* = 17) male mice were anaesthetized with xylazine/ketamine. p38α^Δmyel^ mice do not express p38α in myeloid cells. To induce orthodontic tooth movement, an elastic band (diameter 0.3 mm; Inwaria, Trier, Germany) was inserted between the first and second molar of the left upper jaw by an experienced scientist (CK) as already described [18,19]. Therefore, mice were placed in a custom-made apparatus, which was modified from a previously used rat system [20], and the mandible and tongue were retracted to allow easy access to the oral cavity for interventions. For this purpose, approximately 3 mm of the elastic band were carefully placed between the molars from straight above using mosquito clamps without damaging the surrounding tissue. The elastic band was then shortened at both sides. The maxillary right jaw side was not treated and served as the control side. After induction of orthodontic tooth movement, the food pellets were softened with tap water. Seven days after insertion of the orthodontic appliance, the mice were killed. The upper jaw (WT: *n* = 9; p38α^Δmyel^; *n* = 8) was fixed in 5% formalin overnight and stored in 0.1% formalin until µCT measurements. Tibiae (WT: *n* = 10; p38α^Δmyel^; *n* = 11) were fixed in 4% PFA overnight and then transferred to 70% EtOH until µCT analysis. For RNA analysis, maxillary samples (WT: *n* = 12; p38α^Δmyel^; *n* = 9) were put in liquid nitrogen immediately after removal and stored at −80 °C until isolation.

### 2.2. µCT Analysis

**Figure 1:**
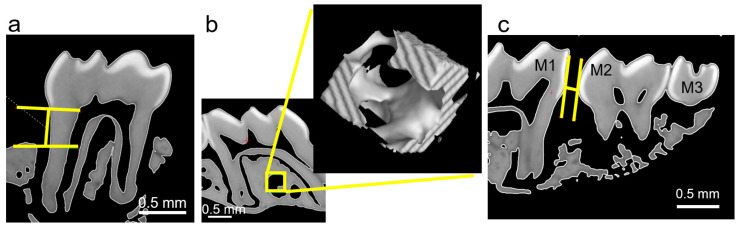
Assessment of periodontal bone loss using the calliper function (**a**), bone density by determining a region of interest (yellow box, (**b**)), which was extracted and enlarged, and orthodontic tooth movement using the calliper funtion (**c**).

µCT measurements (Phoenix vltomelxs 240/180; GE Sensing & Inspection Technologies, Frankfurt am Main, Germany) and image evaluation (Volume Graphics-VG Studio Max, Volume Graphics, Heidelberg, Germany) were performed at the OTH Regensburg. To allow reproducible evaluation, all pictures were aligned within a sagittal layer plane, perpendicular to the occlusal plane and parallel to the palatal suture. Periodontal bone loss at the first molar was measured as the distance between the cemento-enamel junction and the alveolar limbus (Figure 1a). For assessment of bone parameters in the maxillary and tibia samples, a “region of interest” (ROI) with a specified size was defined (Figure 1b and Figure S1). To quantify the extent of orthodontic tooth movement, the smallest distance between the crowns of the first and second molar was determined using the vernier calliper function (Figure 1c).

### 2.3. Histological Analysis

After µCT measurements, tibiae were decalcified, embedded in paraffin, and sectioned at 2 μm. Slides were deparaffinised overnight at 37 °C and hydrogenated. In total, eight sections of each genotype were analysed. 

Safranin Orange G/Light Green stain was used for visualization of osteoblasts in the tibia. The sections were immersed in 3% safranin Orange G solution (T129.1, Carl Roth, Karlsruhe, Germany) for eight min and then rinsed with distilled water. They were then placed in picric acid (P6744-1GA, Sigma-Aldrich, St. Louis, MO, USA) for 10 min, rinsed with H_2_O_d_, and then immersed in 1% acetic acid (3738.1, Carl Roth, Karlsruhe, Germany). Afterwards, sections were incubated for 8 min in 2% light green solution (1.15941.0025, Sigma-Aldrich, St. Louis, MO, USA). The samples were rinsed in 1% acetic acid and dehydrated. Sections were covered with Entellan (1.079.610.500, Merck, Darmstadt, Germany) and digitized under a microscope (IX-50-S8F, Olympus, Shinjuku, Japan). Osteoblasts on the trabeculae were counted and evaluated in relation to the trabecular surfaces using ImageJ (Figure S2a).

To assess osteoclast number, TRAP (tartrate resistant acid phosphatase) stainings were performed. Therefore, slides were placed in a freshly prepared TRAP buffer consisting of 1.64 g sodium acetate (6773.1, Carl Roth, Karlsruhe, Germany) and 23 g of disodium tartrate dihydrate (T110.1, Carl Roth, Karlsruhe, Germany) in 500 mL of H_2_O_dd_ (pH 5) for 10 min at room temperature. Then, the sections were placed in a freshly prepared and prewarmed staining solution consisting of 40 mg Naphtol AS-MX Phosphate Disodium Salt (N5000, Sigma-Aldrich, St. Louis, MO, USA), 4 mL N,N-dimethylformamide (D4551, Sigma-Aldrich, St. Louis, MO, USA), 240 mg Fast Red Violet LB Salt (F3381, Sigma-Aldrich, St. Louis, MO, USA), and 2 mL TritonX-100 (T9284, Sigma-Aldrich, St. Louis, MO, USA) in 200 mL previously prepared TRAP buffer. After incubation at 37 °C for 4 h, the samples were rinsed with H_2_O_d_ and counterstained with hematoxylin solution (51275, Sigma-Aldrich, St. Louis, MO, USA). The sections were covered immediately with Aquatex (1085620050, Merck, Darmstadt, Germany) and digitized under the microscope (IX-50-S8F, Olympus, Shinjuku, Japan; Supplemental Figure S1). The number of TRAP^+^ cells on the trabeculae was determined and evaluated in relation to the trabecular surfaces using ImageJ (Figure S2b).

### 2.4. TRAP5b ELISA

TRACP-5b Elisa (MBS763504, MyBiosource, San Diego, CA, USA) was performed according to the manufacturer’s instructions.

### 2.5. RNA Isolation, Reverse Transcription and RT-qPCR Analysis

A tissue sample containing the first upper left or right molar without the clinical crowns with adjacent periodontal tissue and alveolar bone was macrodissected on a cooling plate. Before RNA isolation, samples were pulverized in a bone mill (frequency 25/s, 2 × 30 s, MM200, Retsch, Haan, Germany). RNA isolation was performed using the PureLink RNA Mini Kit (12183018, Thermo Fischer Scientific, Waltham, MA, USA) according to the manufacturer’s instructions. The recovered RNA was quantified with a nano-photometer (N60, Implen, Munich, Germany). For cDNA synthesis, equal amounts of RNA were added to nuclease free water and mixed with a master mix consisting of 0.1 nmol random hexamer primer (SO142, Thermo Fisher Scientific, Waltham, MA, USA), 0.1 nmol oligo_dT_ primer (SO131, Thermo Fisher Scientific, Waltham, MA, USA), 5 × M-MLV Buffer (M1705, Promega, Madison, WI, USA), 40 nmol dNTP (L785.2, Carl Roth, Karlsruhe, Germany), 200 U reverse transcriptase (M1705, Promega, Madison, WI, USA) and 40 U RNase inhibitor (EO0381, Thermo Fisher Scientific, Waltham, MA, USA). The samples were incubated for 1 h at 37 °C, followed by heat inactivation of the reverse transcriptase at 95 °C for 2 min to. RT-qPCR was performed in a Mastercycler^®^ realplex^2^ (Eppendorf, Hamburg, Germany) with 96-well PCR plates (712282, Biozym Scientific, Hessisch Oldendorf, Germany) and adhesive optical seal film (712350, Biozym Scientific, Hessisch Oldendorf, Germany)). A primer mix containing 0.25 µL each of forward and reverse primer (Table 1), 5 µL Luna Universal qPCR Mix (M3003E, New England BioLabs, Ipswich, MA, USA) and 3 µL RNase-free H_2_O_dd_ (T143, Carl Roth, Karlsruhe, Germany) was prepared for each sample to be examined. To determine the relative gene expression, the formula 2^−ΔΔCq^, with ΔCq = Cq (target gene) − Cq (geometric mean *Eef1a1/Ywhaz*) was used [21].

### 2.6. Data Analysis and Statistics

Statistical analysis was performed using GraphPad Prism version 9.2 (GraphPad Software). First, possible outliers were identified using the ROUT method (Q < 0.1%). Data sets with two groups were analysed for statistically significant differences using a two-tailed Mann–Whitney test. For data sets with more than two groups, the normal distribution of the data was examined with the Shapiro–Wilk test and variance homogeneity with the Brown–Forsythe test. Subsequently, an ANOVA followed by an unpaired *t*-test with Welch correction was performed. If the *p*-value was less than 0.05, the differences were considered statistically significant. No values could be determined for tooth movement on the control side. Therefore, only the OTM side was examined here using a two-sided Mann–Whitney test. Each symbol corresponds to one data point. The horizontal line represents the mean value and the vertical lines represent the standard error.

## 3. Results

### 3.1. Impact of Myeloid p38α/MAPK on Bone Parameters

**Figure 2:**
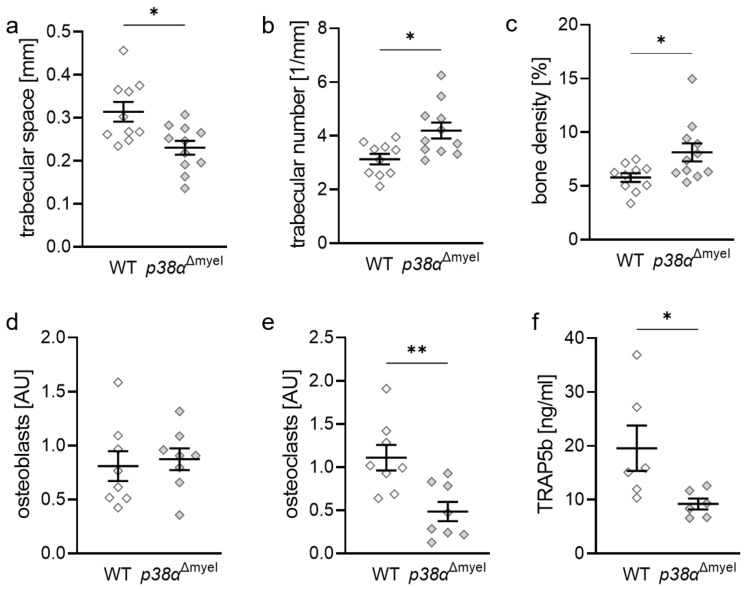
Bone parameter in tibia (*n* ≥ 8) and TRAP5b in serum (*n* = 6) of wildtype (WT) and *p38*^Δmyel^ mice. Trabecular number (**a**), trabecular space (**b**), and bone density (**c**) were obtained by µCT analysis. Osteoblast (**d**) and osteoclast (**e**) number were determined by histological staining. TRAP5b ELISA (**f**) showed reduced levels in serum of *p38α*^Δmyel^ mice. Each symbol respresents one data point. The horizontal line represents the mean value and the vertical lines represent the standard error. Statistics: Mann–Whitney U test; * *p* < 0.05; ** *p* < 0.01.

First, the effect of p38α/MAPK depletion in myeloid cells on bone parameter in the tibia was investigated. Reduced trabecular space (*p* = 0.020; Figure 2a) was detected, while trabecular number (*p* = 0.024; Figure 2b) and bone density (*p* = 0.043; Figure 2c) were increased in *p38α*^Δmyel^ mice. Osteoblast numbers were not changed in mice without p38α in myeloid cells (*p* = 0.645; Figure 2d), while osteoclast numbers were significantly reduced (*p* = 0.005; Figure 2e). To confirm the results for osteoclast number obtained by histological staining, a TRAP5b ELISA was performed and significantly reduced TRAP5b levels in the serum of *p38α*^Δmyel^ mice (*p* = 0.015; Figure 2f) observed, which indicates reduced osteoclast activity in *p38α*^Δmyel^ mice compared to wildtype mice.

### 3.2. Impact of Myeloid p38α/MAPK on the Expression of Inflammatory Genes

**Figure 3:**
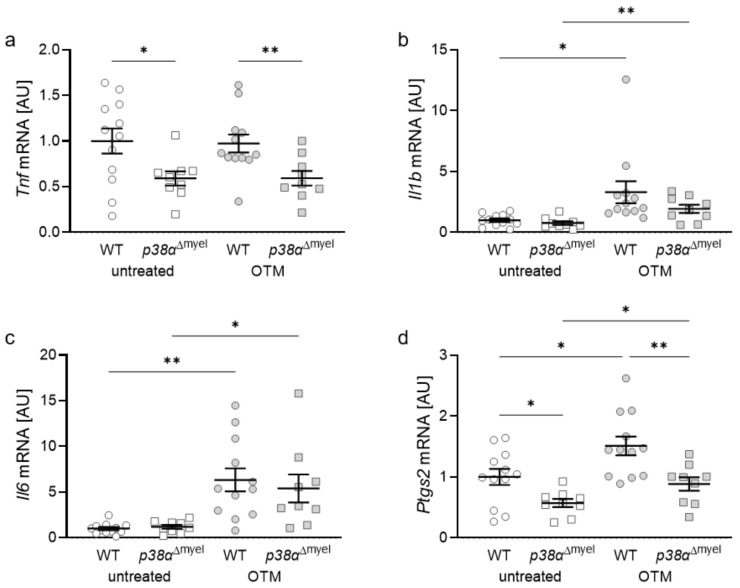
Expression of the inflammatory genes tumor necrosis factor (*Tnf,* (**a**)), interleukin-1b (*Il1b*, (**b**)), interleukin-6 (*Il6*, (**c**)) and prostaglandin endoperoxide synthase-2 (*Ptgs2*, (**d**)) at the orthodontically treated (OTM) and untreated jaw side of wildtype (WT; *n* = 12) and mice without p38α/MAPK in myeloid cells (*p38α*^Δmyel^ mice; *n* = 9). Each symbol respresents one data point. The horizontal line represents the mean value and the vertical lines represent the standard error. Statistics: ANOVA test followed by unpaired *t*-test with Welch’s correction, * *p* < 0.05; ** *p* < 0.01.

Next, the expression of inflammatory genes in the jaw of wildtype and *p38α*^Δmyel^ mice without and with orthodontic tooth movement (OTM) was tested. Gene expression of tumor necrosis factor (*Tnf*) was significantly downregulated in mice without p38α/MAPK in myeloid cells (untreated: *p* = 0.019; OTM: *p* = 0.008), while orthodontic treatment had no impact on *Tnf* gene expression (WT: *p* = 0.871; *p38α*^Δmyel^: *p* = 0.984; Figure 3a). Interleukin-1b (*Il1b;* WT: *p* = 0.028; *p38α*^Δmyel^: *p* = 0.229; Figure 3b) and *Il6* gene expression (WT: *p* = 0.001; *p38α*^Δmyel^: *p* = 0.026; Figure 3c) were significantly increased with orthodontic tooth movement in both tested genotypes. Orthodontic treatment also elevated mRNA levels of prostaglandin endoperoxide synthase-2 (*Ptgs2*) in both genotypes (WT: *p* = 0.019; *p38*α^Δmyel^: *p* = 0.031; Figure 3d). Deletion of p38α/MAPK in myeloid cells reduced *Ptgs2* gene expression without (*p* = 0.010) and with orthodontic treatment (*p* = 0.004).

### 3.3. Impact of Myeloid p38α/MAPK and Orthodontic Treatment on Genes Involved in Bone Remodelling

**Figure 4:**
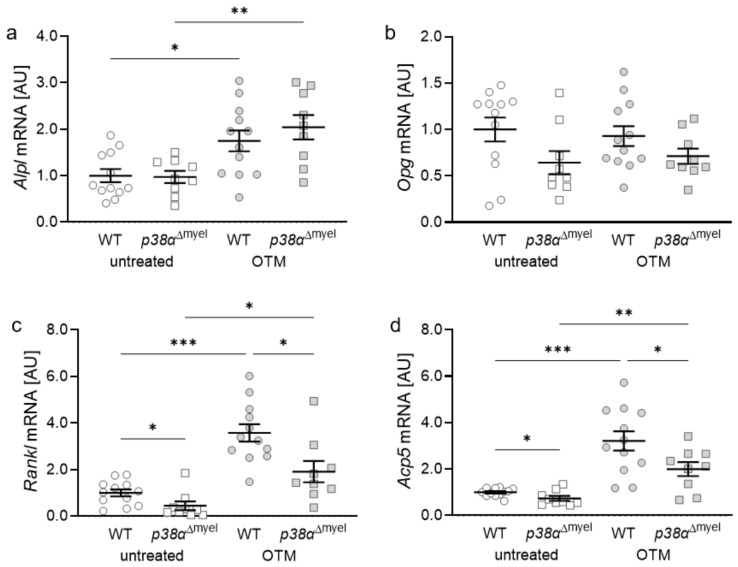
Gene expression of alkaline phosphatase (*Alpl*, (**a**)), osteoprotegerin (*Opg,* (**b**)), receptor activator of NF-kB ligand (*Rankl*, (**c**)), as well as acid phosphatase-5 (*Acp5*, (**d**)) at the orthodontically treated (OTM) and untreated jaw side of wildtype (WT; *n* = 12) and mice without p38α/MAPK in myeloid cells (*p38α*^Δmyel^ mice; *n* = 9). Each symbol respresents one data point. The horizontal line represents the mean value and the vertical lines represent the standard error. Statistics: ANOVA test followed by unpaired *t*-test with Welch’s correction, * *p* < 0.05; ** *p* < 0.01; *** *p* < 0.001.

As bone remodelling is essential for orthodontic tooth movement, expression of genes involved in bone remodelling was investigated. Alkaline phosphatase (*Alpl*) is critically involved in bone formation. *Alpl* gene expression was increased with orthodontic treatment in WT (*p* = 0.011) and *p38α*^Δmyel^ mice (*p* = 0.003; Figure 4a). Depletion of p38α/MAPK had no effect on *Alpl* gene expression with both tested conditions.

The RANKL/OPG system is critical for differentiation of osteoclasts [22,23]. Osteoprotegerin acts as a RANKL (receptor activator of NF-kB ligand) decoy receptor, inhibiting the interaction of RANKL with the RANK receptor on osteoclasts. Neither orthodontic treatment nor deletion of p38α/MAPK had a significant effect on *Opg* gene expression (Figure 4b). In contrast, *Rankl* was increased with orthodontic tooth movement in wildtype (*p* < 0.001) and *p38α*^Δmyel^ mice (*p* = 0.013; Figure 4c), indicating increased osteoclastogenesis in reaction to orthodontic treatment. Deletion of p38α/MAPK in myeloid cells decreased *Rankl* mRNA levels under untreated conditions (*p* = 0.036) and with orthodontic tooth movement (*p* = 0.012). Next, gene expression of acid phoshatase-5 (*Acp5*), which is highly expressed by osteoclasts, was examined. Orthodontic tooth movement increased *Acp5* mRNA levels in wildtype (*p* < 0.001) and *p38α*^Δmyel^ mice (*p* = 0.003; Figure 4d). Corresponding to *Rankl* gene expression, *Acp5* gene expression was downregulated in mice without myeloid p38α/MAPK under both tested conditions (untreated: *p* = 0.046; OTM: *p* = 0.030).

### 3.4. Impact of Myeloid p38α/MAPKs and Orthodontic Treatment on Periodontal Bone Loss and Extent of the Orthodontic Tooth Movement

**Figure 5:**
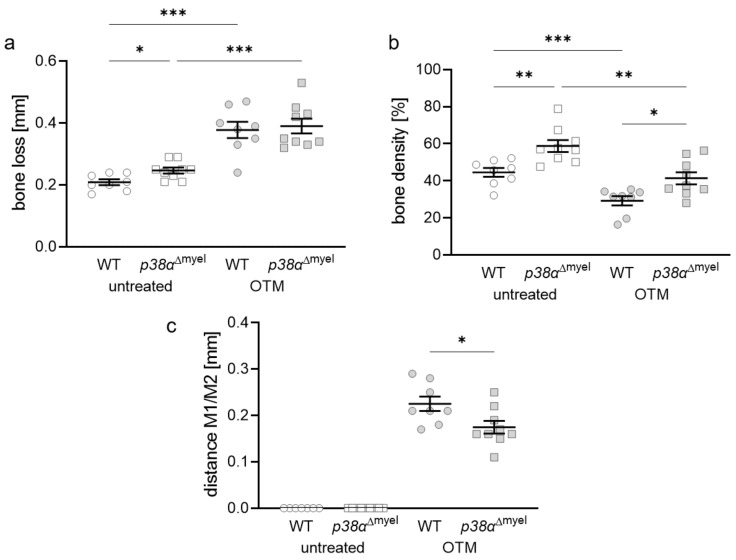
Periodontal bone loss (**a**), bone density (**b**), and orthodontic tooth movement (**c**) as distance between the first (M1) and second (M2) molar at the orthodontically treated (OTM) and the untreated control jaw side in wildtype mice (WT; *n* = 8) and without p38α/MAPK in myeloid cells (*p38*^Δmyel^ mice; *n* = 9). Each symbol respresents one data point. The horizontal line represents the mean value and the vertical lines represent the standard error. Statistics: ANOVA test followed by unpaired *t*-test with Welch’s correction, expect (**c**): Mann–Whitney U test, * *p* < 0.05; ** *p* < 0.01; *** *p* < 0.001.

Finally, the impact of p38α/MAPK in myeloid cells on periodontal bone loss during orthodontic tooth movement was investigated. Periodontal bone loss increased with orthodontic treatment in both tested genotypes (*p* < 0.001) and under untreated conditions in *p38α*^Δmyel^ mice compared to wildtype mice (*p* = 0.013; Figure 5a). Like in the tibia, alveolar bone had a higher bone density in mice lacking p38α/MAPK in myeloid cells at both tested conditions (untreated: *p* = 0.003; OTM: *p* = 0.011; Figure 5b). Bone density was reduced in both genotypes upon orthodontic treatment (WT: *p* < 0.001; *p38α*^Δmyel^: *p* = 0.002). Finally, the extent of orthodontic tooth movement in both genotypes was checked. Corresponding to the higher bone density in mice without p38α/MAPK, the extent of orthodontic tooth movement was significantly reduced (*p* = 0.024; Figure 5c).

## 4. Discussion

Here, the effect of myeloid p38α/MAPK during orthodontic treatment in a mouse model was investigated. Orthodontic tooth movement is ultimately dependent on remodelling processes in the alveolar bone, where force-induced bone formation and resorption processes occur. In mice lacking p38α/MAPK in myeloid cells, reduced tooth movement compared to wildtype mice after orthodontic treatment was observed. This effect may be due to the higher bone density found in *p38α*^Δmyel^ mice in alveolar bone as well as in tibiae, which was also accompanied with reduced trabecular space and increased trabecular number in this tissue. This finding was in line with Cong et al., who reported a higher bone density in two-month-old mice lacking p38α/MAPK [12]. The higher bone density was explained by a reduced osteoclast number in *p38α*^Δmyel^ mice, as this leads to reduced bone resorption. Accordingly, lower osteoclasts numbers in tibiae and reduced *Acp5* gene expression in periodontal tissue in *p38α*^Δmyel^ compared to wildtype mice were detected. Next to other genes osteoclast-specific genes like *cathepsin K* or *calcitonin receptor*, *Acp5* is mainly expressed by osteoclasts. Therefore, the downregulated *Acp5* gene expression could be hinting at a reduced osteoclast number in the alveolar bone of *p38α*^Δmyel^ mice, resulting in reduced bone resorption. 

Orthodontic treatment provokes a sterile inflammation in the periodontal ligament. Macrophages were shown to react to mechanical stress with increased secretion of inflammatory factors like tumor necrosis factor, interleukin-6 (IL6), and prostaglandin E2 [6]. Here, no effects of orthodontic tooth movement on *Tnf* gene expression were detected, while *Il6* and prostaglandin endoperoxide synthase 2 (*Ptgs2*) were upregulated on the orthodontically treated maxillary jaw side, which was in line with several previous studies [19,24,25]. However, involvement of p38α/MAPK in this mechanically-induced upregulation has not been tested so far. Deletion of p38α/MAPK in myeloid cells had no impact on *Tnf*, *Il1B* or *Il6* gene expression, but gene expression of *Ptgs2* was reduced without and with orthodontic treatment. However, in mesangial cells, p38 was reported to be involved in upregulation of PG-E2 [26].

Bone formation is mainly regulated by osteoblasts. Bone formation is associated with osteoblast proliferation and differentiation, and with the expression of numerous genes including alkaline phosphatase (*Alpl*). *Alpl* is involved in matrix mineralization. In the present study, an inducing effect of *Alpl* gene expression in periodontal tissue on orthodontic tooth movement was demonstrated. These data are consistent with previous studies on periodontal ligament fibroblasts, which showed an increase in *Alpl* gene expression and protein secretion upon mechanical stress [9,27]. Deletion of p38α/MAPK in myeloid cells had no effect on *Alpl* gene expression in periodontal tissue. This effect may have been masked by the inducing effect of periodontal ligament fibroblasts. Cong et al. performed co-culture experiments with mesenchymal stem cells (MSCs) and monocytes to test whether the deletion of p38α/MAPK in monocytes had an effect on the differentiation of MSCs to ALPL-expressing osteoblasts [12]. They detected reduced ALPL-positive MSCs after co-cultivation with p38α/MAPK-deficient monocytes, and concluded a reduced capacity in supporting MSC differentiation. In contrast to Cong et al., we detected no significant reduced osteoblast number in the tibia [12]. 

Osteoclast differentiation is dependent on the ratio of RANKL to OPG [22,28]. Interaction of RANKL with the RANK receptor on osteoclast precursor cells led to the differentiation of mature, bone-resorbing osteoclasts. No influence of orthodontic tooth movement or deletion of p38α/MAPK on *Opg* gene expression was observed. This was in line with previous studies reporting that orthodontic treatment did not affect *Opg* expression in periodontal tissue [19,24,25]. Periodontal ligament fibroblasts react to mechanical strain in vitro with a reduction of *Opg* gene expression [27]. In in vitro experiments using osteoblasts, associations between p38 and OPG secretion were detected [29]. In contrast to *Opg*, *Rankl* gene expression was increased by orthodontic treatment in both wildtype [19,24] and *p38α*^Δmyel^ mice. Gene expression of *Rankl* was increased, when p38α/MAPK was deleted in myeloid cells in periodontal tissue on both the control and orthodontically-treated maxillary jaw sides. Gene expression of osteoclast specific *Apc5* was altered according to *Rankl* gene expression in the investigated groups. These data were in line with previous studies conducted in RAW264.7 macrophages [30]. The authors observed impaired osteoclastogenesis after treatment with recombinant RANKL and a p38 inhibitor. 

In addition to the observations that could be made here, the conducted study also has limitations. RNA analysis was performed from the surrounding dental tissue, which consists mainly of periodontal ligament fibroblasts in addition to myeloid cells. Therefore, possible effects on gene expression of myeloid cells could be masked by gene expression of periodontal ligament fibroblasts. Therefore, further in vitro studies should be performed using myeloid cells with modulated p38 activity under concomitant mechanical stress. A study in mice can only give an impression of what is happening in humans upon orthodontic treatment. Furthermore, only one time point was investigated and only young mice were used. As Cong et al. described a tremendous age effect, it would be interesting to obtain also data from old mice, especially in view of the more frequent adult treatments in orthodontics [12].

## 5. Conclusions

This study provides new insights into the regulatory role of myeloid p38α/MAPK during orthodontic treatment. As demonstrated, p38α/MAPK derived from myeloid cells had various effects on processes that are involved in orthodontic tooth movement, namely inflammation and osteoclastogenesis. Therefore, it is concluded that myeloid p38α/MAPK impacts on bone density, thereby affecting the extent of tooth movement. Myeloid p38α/MAPK thus play a regulatory role during orthodontic tooth movement by modulating osteoclastogenesis. 

## Figures and Tables

**Table 1:** Primer sequences of used reference (*Eef1a1/Ywhaz*) and target genes.

Gene	Gene Name	5′-*Forward* Primer-3′	5′-*Reverse* Primer-3′
*Acp5*	Acid Phosphatase 5, Tartrate-Resistant	ATACGGGGTCACTGCCTACC	TCGTTGATGTCGCACAGAGG
*Alpl*	Alkaline Phosphatase	GGGTACAAGGCTAGATGGC	AGTTCAGTGCGGTTCCAGAC
*Eef1a1*	Eukaryotic Translation Elongation Factor 1 Alpha 1	AAAACATGATTACAGGCACATCCC	GCCCGTTCTTGGAGATACCAG
*Il1b*	Interleukin 1 beta	GTGTAATGAAAGACGGCACACC	ACCAGTTGGGGAACTCTGC
*Il6*	Interleukin 6	ACAAAGCCAGAGTCCTTCAGAG	GAGCATTGGAAATTGGGGTAGG
*Opg*	Osteoprotegerin	CCTTGCCCTGACCACTCTTAT	CACACACTCGGTTGTGGGT
*Ptgs2*	Prostaglandin-Endoperoxide Synthase 2	TCCCTGAAGCCGTACACATC	TCCCCAAAGATAGCATCTGGAC
*Rankl*	Receptor Activator of NF-κB Ligand	AAACGCAGATTTGCAGGACTC	CCCCACAATGTGTTGCAGTTC
*Tnf*	Tumor Necrosis Factor	ACAAGCCTGTAGCCCACGTC	TTGTTGTCTTTGAGATCCATGCC
*Ywhaz*	Tyrosine 3-Monooxygenase/Tryptophan 5-Monooxygenase Activation Protein Zeta	AATGCTTCGCAACCAGAAAGC	TGGTATGCTTGCTGTGACTGG

## Data Availability

All datasets are publicly available either as Supplementary Material to this article or upon request from the corresponding author.

## Supplementary Materials

The following supporting information can be downloaded at: https://www.mdpi.com/article/10.3390/jcm11071796/s1, Figure S1: For assessment of bone parameters in tibia samples, a “region of interest” (ROI) with a specified size was defined; Figure S2: Osteoblasts (a) and osteoclasts (b) were counted and evaluated in relation to the trabecular surfaces.
