# Supplementary material for: Impact of Myeloid p38α/MAPK on Orthodontic Tooth Movement
Source: J Clin Med. 2022 Mar 24;11(7):1796. doi: 10.3390/jcm11071796 (PMC9000068; doi:10.3390/jcm11071796)
Supplement: Supplementary file 1 [file jcm-11-01796-s001.zip › jcm-1623952-supplementary.pdf]

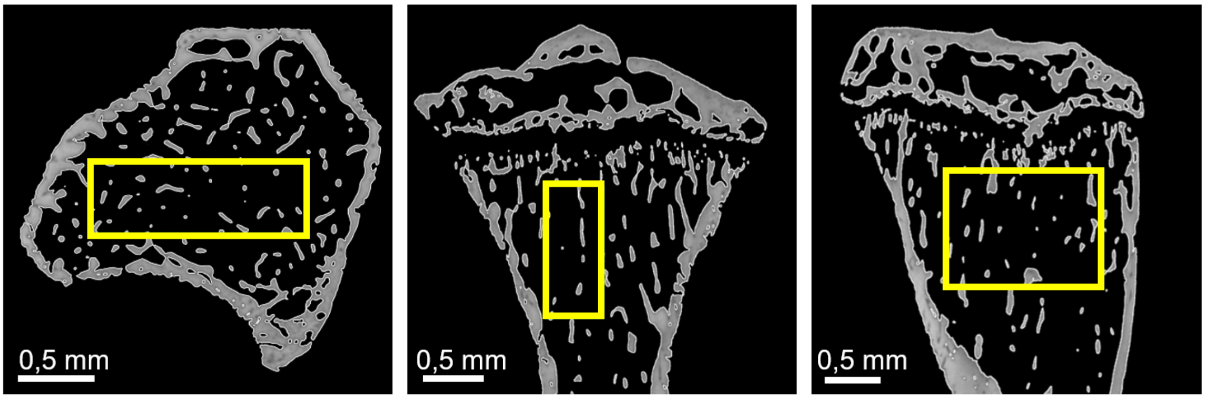

Figure S1. For assessment of bone parameters in tibia samples a "region of interest" (ROI) with a specified size was defined.

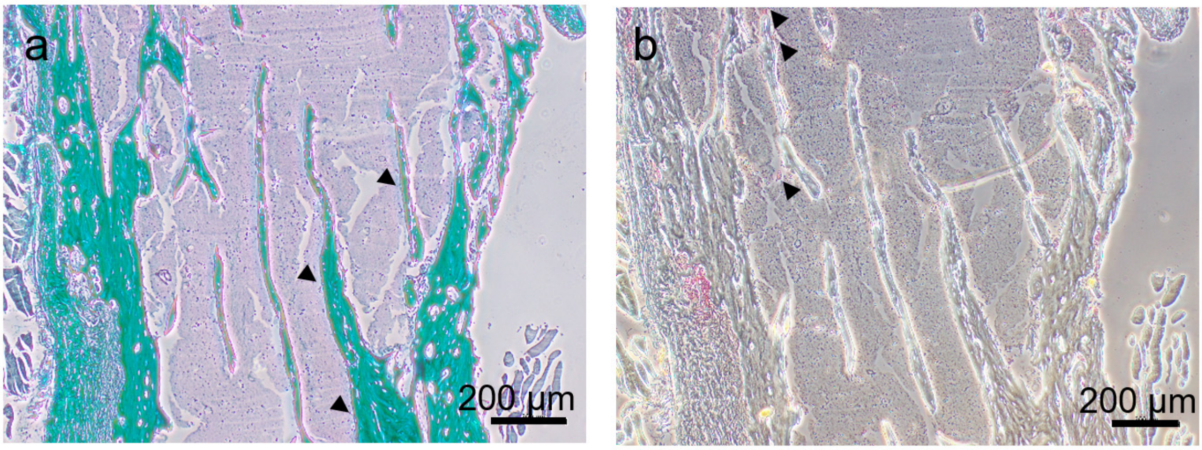

Figure S2. Osteoblasts (a) and osteoclasts (b) were counted and evaluated in relation to the trabecular surfaces.
